# Characteristics of patients with osteonecrosis of the jaw with oral versus intravenous bisphosphonate treatment

**DOI:** 10.1186/s40902-021-00310-w

**Published:** 2021-07-08

**Authors:** Seung-Hun Lee, So-Young Choi, Min-Su Bae, Tae-Geon Kwon

**Affiliations:** Department of Oral and Maxillofacial Surgery, School of Dentistry, Institute for Translational Research in Dentistry, Kyungpook National University, 2177 Dalgubeol-daero, Jung-gu, Daegu, 41940 Republic of Korea

**Keywords:** Osteonecrosis, Jaw, Bisphosphonate, Intravenous, Oral, Medication

## Abstract

**Purpose:**

This retrospective study was aimed to evaluate the clinical characteristics and treatment outcomes in patients with osteonecrosis of the jaw who were receiving oral versus intravenous (IV) bisphosphonate (BP).

**Materials and methods:**

This retrospective study enrolled subjects who had been diagnosed with medication-related osteonecrosis of the jaw (MRONJ) during the period from July 2010 to June 2014. Information regarding the following demographic and clinical characteristics was collected: demographic data, administration route and type of BP, duration of BP medication, primary disease, number of involved sites, location of the lesion, number of surgeries, outcome of treatments, and laboratory test. All the patients were divided into oral and IV BP groups; and the between-group differences were compared.

**Results:**

Total 278 patients were divided into two groups as per the route of BP administration. The proportion of oral BP-related MRONJ group were more dominant over IV BP group (oral BP, n = 251; IV BP, n = 27). In the IV BP group, the average dosing duration (31.4 months) was significantly shorter than that in the oral BP group (53.1 months) (P < 0.001). The average number of involved sites in the oral BP group (1.21 ± 0.48) was smaller than that in the IV BP group (1.63 ± 0.84) (P < 0.001). The average number of surgeries was higher in the IV BP group (1.65 ± 0.95) as compared to that in the oral BP group (0.98 ± 0.73) (P < 0.001). Outcome after the surgery for MRONJ after IV BP was poor than oral BP group.

**Conclusion:**

IV administration of BP causes greater inhibition of bone remodeling and could lead more severe inflammation. Therefore, even if the duration of IV administration of BP is shorter than that of oral BP, the extent of the lesion could be more extensive. Therefore, the result suggests that the MRONJ after IV BP for cancer patients needs to be considered as different characteristics to oral BP group for osteoporosis patents.

## Background

Bisphosphonates (BP) are widely used in the treatment of osteoporosis and other metabolic disease as well as malignant tumors. Oral BP are approved for the treatment of osteoporosis, osteopenia, and other rare bone disease including Paget disease and osteogenesis imperfect [1]. Intravenous (IV) BP are used to treat patients with bone metastasis from malignant tumor, hypercalcemia of malignancy, and lytic lesions in multiple myeloma [2]. However, adverse effects, such as medication-related osteonecrosis of the jaw (MRONJ) have been reported. After the first publication regarding MRONJ (2003) by Marx [3], the long-term use of BP or high-dose intravenous (IV) administration was considered a risk factor for osteonecrosis of the jaw (ONJ). BP exert antiresorptive effects via the inhibition of osteoclast differentiation and function. Some reports have shown soft tissue toxicity that causes increased apoptosis or decreased proliferation of epithelial cells after exposure to BP [5]. Generally, IV BP, such as zoledronate possess greater potency than oral BP, resulting in more rapid binding and greater accumulation in the bone [6]. There are several risk factors for the development of MRONJ and can be divided into the following 4 categories: (1) medication-related risk factors, (2) local factors, (3) systemic factors, and (4) genetic factors [4]. Each category includes a subgroup of risk factors, such as dentoalveolar surgery, corticosteroid use, diabetes mellitus, type of BP, route of BP administration, and duration of medication. MRONJ tends to occur more frequently in patients on IV BP than in those on oral BP [4, 7, 8]. The antiresorptive capacity of IV BP is higher than that of oral BP. In patients with ONJ after IV BP, it had been shown that there was significant decrease in bone turn over marker [9–11]. MRONJ can be managed with conservative treatment using antibiotics, minimally invasive surgical debridement, or radical surgical resection based on the severity of the lesion. The treatment strategy should be selected according to the MRONJ staging [4]. However, few studies have studied the MRONJ pattern and treatment outcomes as per the route of drug administration using an adequate sample size. It had been suggested that ONJ is usually less aggressive and is associated with better treatment response after oral BP administration than after IV BP [11].

According to a clinicopathological investigation of MRONJ, oral BP was not significantly different from IV BP in terms of the healing time or pathological findings of the lesions [12]. Conservative surgical treatments, such as sequestrectomy and curettage, are generally effective in the treatment of MRONJ after oral BP administration [13]. It was also suggested that favorable results had been achieved after radical resection for IV BP-related ONJ patients but had variable treatment outcomes [14].

Shintani et al. [15] analyzed the prognosis of 59 MRONJ patients (29 patients of oral BP versus 30 patients with IV BP administration). Hallmer et al. [16] compared the demographic figures and outcomes of 24 patients who were given oral BP and 31 who were given IV BP. They analyzed the treatment results and clinical features after MRONJ treatment and compared the difference between those who received oral BP and IV BP. Both the reports showed that MRONJ associated with oral BP showed better treatment outcomes than that associated with IV BP. In these studies, there were no comparisons of the laboratory test and the severity of lesions; further, the number of patients who underwent surgery was insufficient. Moreover, there is insufficient data regarding post-treatment results, such as number of reoperation after initial treatment. Bermudez-Bejarano et al. [17] reviewed the literature regarding MRONJ treatment and classified the treatment protocol for 7 categories. They evaluated the outcomes in order to analyze the efficacy of different treatment protocols for the lesion. However, the statistical analysis of the outcomes as per the different protocols could not be performed owing to the limited number of the subjects and variability in the protocols. Therefore, the disease characteristics as per route of administration warrants further investigation.

The present retrospective study was designed in order to evaluate the clinical characteristics and treatment outcomes of ONJ related with oral and IV BP administration. We focused on the difference in the lesion patterns and number of surgeries required until treatment completion. We further determined whether there was a difference in the prognosis based on the administration method by collecting data from a larger number of patients.

## Materials and methods

### Subjects

This retrospective study was conducted at the Kyungpook National University Hospital, and the study subjects were enrolled from July 2010 to June 2014. Among patients diagnosed with MRONJ, those with an insufficient follow-up period or lack of clinical, radiological, or laboratory data were excluded. All the study subjects were followed up for at least 6 months after MRONJ diagnosis. For a clear comparison based on the administration route, patients diagnosed with MRONJ who were administered both oral and intravenous BP concurrently were excluded. This study received ethical approval from the Institutional Review Board (KNUDH IRB 2021-03-01-00).

### Assessment of demographic and clinical data

According to the American Association of Oral and Maxillofacial Surgeons (AAOMS) position paper (2014) [4], patients can be diagnosed with MRONJ if all the following characteristics are present: (1) current or previous treatment with antiresorptive or antiangiogenic agents, (2) exposed bone or bone that can be probed through an intraoral or extraoral fistula in the maxillofacial region that has persisted for > 8 weeks, and (3) no history of radiation therapy to the jaws or obvious metastatic disease to the jaws.

Demographic and clinical features, such as patients’ age, sex, primary cause of BP treatment, type and duration of BP medication, and staging of MRONJ were analyzed. Staging of the MRONJ was defined in AAOMS position paper (2014) [4] as follows: stage 0, no clinical evidence of necrotic bone, but non-specific clinical findings, as well as radiographic changes and symptoms; stage 1, exposed and necrotic bone/fistulae that can be probed to the bone, asymptomatic, and no evidence of infection; stage 2, exposed and necrotic bone/fistulae that can be probed to the bone, associated with infection; stage 3, exposed and necrotic bone/fistulae that can be probed to the bone, associated with infection and additional complications.

### Location of the lesions and treatment outcomes

The patients were treated as per the AAOMS guidelines (2014) [4]. Once diagnosed with MRONJ, they first underwent conservative treatment, including oral antimicrobial rinses, such as 0.12% chlorhexidine, or combination therapy with antibiotics. If the clinical symptoms reduced or disappeared with conservative combination therapy, surgery was not performed. However, if there was no reduction in the signs and symptoms after conservative treatment or if sequestrum was observed on clinical and radiological examination, sequestrectomy and curettage were performed under local or general anesthesia. We continued to follow-up patients who did not undergo surgery, and treatment was terminated when the clinical signs and symptoms reduced.

The number of involved sites was counted as per previous studies, and the maxillomandibular structure was divided into the following 6 areas: anterior, right posterior maxilla, left posterior maxilla, anterior posterior mandible, right posterior mandible, and left posterior mandible [16, 18]. In order to determine the prognosis of these patients, those who had undergone surgery were analyzed for healing patterns and number of surgeries. Among the patients who underwent surgery, those with recurrence or no improvement were re-operated. The number of reoperations was evaluated as a measure of treatment prognosis. The number of surgeries is the most important index used for evaluating the prognosis.

After initial surgery, the healing pattern of the wound was classified into the following 3 categories: “good”, “moderate”, and “poor”. If there was no clinical symptom and the exposed bone was completely covered by intact mucosa, it was defined as “good”. If there was reduced bone exposure and pain, it was defined as “moderate”. “Poor” healing pattern was defined as that wherein pain and bone exposure were persistent. Even postoperatively, persistent bone exposure or more severe inflammation and bone destruction were considered to indicate treatment failure.

### Laboratory findings at the time of MRONJ diagnosis

Laboratory data at the time of MRONJ diagnosis were evaluated; bone resorption markers (c-telopeptide of collagen type 1, CTX) and inflammatory activity markers (erythrocyte sedimentation rate, ESR; C-reactive protein, CRP) were tested using the same method as reported previously [19].

### Statistical analyses

Statistical analyses were performed with SPSS Statistics program (ver. 21.0; IBM Corp., Armonk, NY, USA). In order to evaluate the difference between the oral and IV BP groups in terms of the clinical features and prognosis, independent T test had been utilized. Chi-squared test was used to compare the categorical variables, such as the MRONJ stage. A P value < 0.05 was considered to be statistically significant.

## Results

### Demographic and clinical data of the patients

Total 294 patients were clinically, radiologically, and historically diagnosed with MRONJ. Of these subjects, 16 patients who received both oral and IV BP were excluded. Finally, 278 patients were enrolled in the study (oral BP, n = 251; IV BP, n = 27). The patients in the oral BP group (73.2 ± 7.5 years) were significantly older than those in the IV BP group (64.1 ± 7.7 years) (p < 0.001). Females more dominant in the oral BP group (96.4%) than IV BP group (70.4%) (p < 0.001). The primary cause for administering BP in the oral BP group was osteoporosis (n = 251), while that in the IV BP group was cancer (n = 27). Among the patients in the oral BP group, 231 (92.0%) were treated with single BP therapy, and 20 (8.0%) were treated with combination BP therapy. Alendronate was the most common BP agent in the single-use therapy group (n = 141, 61.0%). In the IV BP group, most patients (n = 32, 94.1%) received single BP therapy. Zoledronate was the predominant BP agent in the IV BP group (n = 24, 75.0%). The average medication duration was 53.1 ± 36.6 months in the oral BP group and 31.4 ± 20.4 months in the IV BP group. Thus, the dosing duration was significantly shorter in the IV BP group (p < 0.001).

Stage-3 MRONJ was the predominant clinical stage in the oral BP group (60.2%, n = 151). However, in the IV BP group, stage 2 patients were most common (51.9%, n = 14). There was no significant correlation between the administration method and the MRONJ stage on chi-square analyses. Although the severity of MRONJ appeared to be greater in the oral BP group, the difference was not statistically significant (p = 0.079) (Table 1).

**Table 1:** Comparison of the demographic and clinical findings of the oral BP and IV BP groups

Measurements	Oral BP Mean ± SD	IV BP Mean ± SD	Difference *p* value
**Number of patients**	n = 251	n = 27	
**Age (years)**	73.2 ± 7.5 (min 40, max 90)	64.1 ± 7.7 (min 51, max 80)	< .001
**Sex (female, %)**	n = 242 (96.4%)	n = 19 (70.4%)	< .001*
**Primary cause of BP medication**			
Osteoporosis	251	–	
Malignant disease	–	27	
Multiple myeloma	–	15	
Breast cancer	–	10	
Prostate cancer	–	2	
**BP medication**			
Alendronate (weekly, oral)	141 (56.2%)	–	
Risedronate (weekly, or monthly, oral)	47 (18.7%)	–	
Ibandronate (monthly, oral)	36 (14.3%)	–	
Used multiple oral BP	20 (8.0%)	–	
Pamidronate (monthly, IV)	–	1 (3.7%)	
Zolendronate (monthly, IV)	–	24 (88.9%)	
Used multiple IV BP	–	2 (7.4%)	
**Duration of medication (months)**	53.1 ± 36.7	31.4 ± 20.4	.003
**MRONJ clinical stage**			.079*
Stage 0	9 (3.6%)	1 (3.7%)	
Stage 1	6 (2.4%)	2 (7.4%)	
Stage 2	84 (33.5%)	14 (51.9%)	
Stage 3	152 (60.5%)	10 (37.0%)	

Inter-group differences were analyzed with independent t test; chi-squared test* was used for categorical variables

### Characteristics of the MRONJ lesions

The average number of involved sites in the MRONJ patients was 1.23 ± 0.51. In the oral BP group, the average number of involved sites was 1.21 ± 0.48, while that in the IV BP group was 1.63 ± 0.84. Significantly more sites were involved in the IV BP group than oral BP group (p < 0.001). Most patients in the oral BP group showed the involvement of a single site (n = 206, 82.1%). However, in the IV BP group, the ratio of single site (n = 15, 55.6%) was smaller than in oral BP group, and the ratio of more than two sites was relatively higher (two sites n = 8; 29.6%). In both groups, the mandible was the dominant area for MRONJ development. Occurrence in both mandible and maxilla tended to be more pronounced in the IV BP group. This difference between IV versus PO BP groups in location of the lesion was statistically significant (p = 0.012) (Table 2).

**Table 2:** Characteristics of the lesion and treatment results

Variables	Oral BP Mean ± SD	IV BP Mean ± SD	Difference *p* value
**Number of involved sites**			
1 site	206 (82.1%)	15 (55.6%)	
2 sites	37 (14.7%)	8 (29.6%)	
3 sites	8 (3.2%)	3 (11.1%)	
4 sites	206 (82.1%)	1 (3.7%)	
Average number of lesions	1.21 ± 0.48	1.63 ± 0.84	< .001
**Location of the lesion**			**.012**
Maxilla	74 (29.5%)	5 (18.5%)	
Mandible	161 (64.1%)	16 (59.3%)	
Both	16 (6.4%)	6 (22.2%)	
**Number of the surgery**			
0	7 (2.8%)	–	
1	205 (81.7%)	15 (55.6%)	
2	29 (11.5%)	7 (25.9%)	
3	8 (3.2%)	2 (7.4%)	
> 4	2 (0.8%)	3 (11.1%)	
**Recurrent rate**	39/251 (15.5%)	12/27 (44.4%)	< .001^*^
**Average number of the surgery**	0.98 ± 0.73	1.65 ± .095	< .001
**Outcome after initial surgery**			< .001*
Good	205 (81.7%)	12 (44.4%)	
Moderate	44 (17.5%)	13 (48.1%)	
Poor	2 (0.8%)	2 (7.4%)	

Inter-group differences were analyzed with independent t test; chi-squared test* was used for categorical variables

### Treatment modality

In the oral BP group, 244 out of 251 patients had undergone surgery, and all patients in the IV BP group had undergone surgery. The number of operations reflected repetitive surgery and this was regarded as an important indicator of treatment prognosis.

In the oral BP group, the signs and symptoms reduced in 2.8% (n = 7) of the patients with only conservative treatment. Most patients had undergone only a single surgery (81.7%, n = 205) and 11.5% (n = 29) and 3.2% (n = 8) of the patients had undergone 2 and 3 surgeries, respectively. One patient each who undergone 5 and 6 surgeries; however, these cases account for a very small proportion of the population.

In the IV BP group, all patients had undergone surgery, and more subjects in this group had undergone 2 times of surgeries (44.4%, n = 12). Among those who had undergone surgery, the average number of surgeries was significantly more in the IV BP group than in the oral BP group (1.65 ± 0.95 vs. 0.98 ± 0.73) (p < 0.001). We found that 39 out of 251 (15.5%) patients in the oral BP group and 12 out of 27 (44.4%) patients in the IV BP group had recurrence following conservative treatment and first surgery (p < 0.001).

The most common healing pattern after initial surgery was “good” in oral BP group (n = 205, 81.7%). However, in the IV BP group, “good” state of healing was only observed in 44.4% (n = 12) and “moderate” outcome was the most common, observed in 48.1% (n = 13) of the patients. “Poor” outcome was significantly more common in the IV BP group (7.4%, n = 2) than in the oral BP group (0.8%, n = 2) (p < 0.001) (Table 2).

### Laboratory findings

The average CTX of the oral BP group was significantly higher than that of the IV BP group (189 ± 151 pg/mL vs. 134 ± 78 pg/mL, p = 0.031). In this study, the mean ESR was slightly higher in the IV BP group (49.33 ± 27.72) as compared to that in oral BP group (40.99 ± 23.97) but did not reach statistical significance (p = 0.516). In contrast, the average CRP level was significantly higher in the IV BP group (2.13 ± 3.08) than in the oral BP group (1.01 ± 1.59) (p < 0.001) (Table 3).

**Table 3:** Laboratory findings

Laboratory test	N (oral/IV BP)	Oral BP	IV BP	P value
CTX (pg/mL)	100/18	189.1 ± 151.3	133.7 ± 78.3	.031
ESR (mm/h)	170/19	40.99 ± 23.97	49.33 ± 27.72	.516
CRP (mg/dL)	170/19	1.01 ± 1.59	2.13 ± 3.08	.001

Inter-group differences were analyzed with independent t test

## Discussion

It is known that stronger the potency of BP, higher the risk of developing MRONJ. In general, the incidence of MRONJ with IV medication is significantly higher than that with oral medication. Oral BP had a relatively smaller effect on the osteoclast function because oral BP has a lower absorption rate (10%) in the gastrointestinal tract and is excreted largely unchanged by the kidneys [4, 20, 21]. It had been reported that the MRONJ incidence with a high dose of IV BP is significantly higher than oral BP [22]. AAOMS position paper on MRONJ also noted that patients with cancer treated with IV BP had a high incidence of ONJ, whereas the risk of developing ONJ in osteoporotic patients exposed to oral or IV BP remained very low [4]. Thomas et al. [23] reported that among patients with cancer who were exposed to IV BP, the ONJ risk ranged from 0 to 6.7%, and the chances of developing ONJ in patients with cancer exposed to an oral BP was 0.7%. Systematic review on the treatment and outcome of MRONJ concluded that IV BP is more frequently associated with ONJ than oral BP [9]. AAOMS position paper suggested that the incidence of ONJ in oral BP users is very low [4]. However, long-term administration of oral BP also can significantly suppress bone turnover and ONJ is one of the major concern for the osteoporosis patients with oral BP [24]. Recent national survey in Japan (2018) [25] showed that proportion of ONJ related with oral BP was increased compared to 2014 data [7], which is nearly similar to IV BP-related ONJ. Taiwanese population showed higher incidence of 82 per 100,000 person who had been received alendronate [26]. A report from Sweden suggested that 67 cases per 100,000 patient-years [27]. According to the Korean data, ONJ incidence was suggested as 21 per 100,000 person in osteoporosis patients [28], which is comparable to 28 per 100,000 person-years of oral BP treatment in data from the USA [1]. The previously reported incidence was much higher than number of reported ONJ cases after alendronate treatment for osteoporosis of approximately 0.7 cases per 100,000-year exposure from Merck in 2006 [29]. In our institute, the ONJ caused by oral BP administration was predominatly higher (90.3%) than IV BP group (9.7%). It is also reported at the nationwide survey on clinical department in Korea (2013) and showed that 78.7% of reported BRONJ cases were related with oral BP and much higher than IV BP (21.3%) [30]. Although the incidence of ONJ is low in Korea [28], a long-term administration and increased cumulative dose of BP might be attributed to increased proportion of the ONJ cases in oral BP group in our study.

The important finding of this study was the difference in the clinical features and treatment prognosis of MRONJ related with oral versus IV BP. In this study, the oral BP group was likely to develop at older age (73.2 years) than the IV BP group (64.1 years). Our results confirm previous reports by Shintani et al. [15] who reported similar pattern of mean age; oral BP 76.6 years versus IV BP 67.3 years. Similarly, Hallmer et al. [16] reported that osteoporosis with oral BP were older than those with cancer treated with IV BP. The duration of medication in the IV BP group was significantly shorter than that in the oral BP group. In general, IV BP has a considerably higher absorption rate and potency in the body than oral BP; therefore, MRONJ is more likely to occur even if the duration of IV medication is shorter than that for oral BP.

The development of MRONJ with oral BP requires a long period of exposure (53.1 months) than IV BP (31.4 months). Lo et al. reported a higher prevalence of MRONJ (0.21%) in patients treated with oral BP for > 4 years as compared to that in those who were treated for < 2.5 years [1]. As reported in a previous literature, the longer duration of BP therapy is one of risk factor of MRONJ, and the risk appears to be higher after 3 years of treatment [31].

The number of involved sites was also higher in the IV BP group than in the oral BP group (1.63 ± 0.84 vs. 1.21 ± 0.48). This result implies the stronger effect of IV BP than oral BP in developing ONJ. MRONJ appears to be more likely to affect the mandible and maxilla than the other parts of the skeleton. The jaws are the only bones that are in contact with the outside frequently and are subject to repeated micro-trauma through the presence of teeth and the forces associated with mastication. Moreover, the turnover of the alveolar bone is 10 times as much as that of the long bones [28, 32]. The mandible was affected by MRONJ more commonly than the maxilla. This could be attributed to the decreased vascularity of the mandible, and the existing local conditions could contribute to MRONJ. This distribution is similar to that reported previously. Aljohani et al. [33] also showed that the mandible was the most common MRONJ site, followed by the maxilla and both. Haller et al. [34] also reported that most lesions were located in the mandible (75%). In a similar manner, in our study, the incidence of MRONJ in the mandible was higher in both the groups. However, the tendency to occur more in both jaws was greater in the IV BP group.

In the present study, the evaluation of treatment prognosis was based on the number of surgeries. Reoperation was performed if there was no improvement or recurrence after conservative and surgical treatment. Therefore, the number of operations could be used as a measure for evaluating patient prognosis. The average number of surgeries in patients treated with IV BP (1.65 ± 095) was higher than that in those given oral BP (0.98 ± 0.73). In our experience, most of the patients with oral BP underwent surgery with sequestrectomy and surgical curettage rather than radical surgical resection. However, definitive sequestra formation had not been existed in IV BP-related ONJ and there was difficulty in establishing the margin of the lesion.

It had been proposed that ONJ after oral BP is a rare and real entity that is less frequent, less severe, more predictable, and more responsive to treatment than IV BP-induced osteonecrosis [35]. Other report showed that > 90% of the patients treated with oral BP could be cured. However, only 50% of those treated with IV BP showed no improvement [15]. IV BP treatment generally causes more advanced and extensive BRONJ and is less sensitive to conservative treatment than oral BP [36]. Similar to the previously reported results, the treatment prognosis of MRONJ for patients with oral BP was better than that for patients with IV BP in our study.

Reich et al. [37] categorized the postoperative results into 4 groups. Similarly, in this study, we divided the treatment outcomes into 3 groups, as “good”, “moderate”, and “poor” to evaluate the postoperative prognosis. In the oral BP group, “good” outcome was the most common (81.7%, n = 205) after initial surgery; thus, the treatment outcomes were relatively favorable. However, in the IV BP group, the proportions of patients with “moderate” or “poor” outcomes (55.5%, n = 15) were higher, indicating that reoperation was required more frequently.

Serum CTX refers to the examination of C-terminal cross-linking telopeptide of type I collagen. Type I collagen is a structural organic compound that accounts for 98% of the total protein in the bone. Telopeptide fragment is a derivative from which the main cross-linking chain proceeds with bone resorption by osteoclasts. The serum level of the telopeptide fragment is proportional to the level of osteoclast activity at the time of blood collection. Therefore, serum CTX is considered most relevant for bone replacement. The normal level of serum CTX is usually > 300 pg/mL, and in most patients, it ranges from 400 pg/mL to 550 pg/mL. An imbalance in bone remodeling resulting from the suppression of osteoclastic activity is a major factor related to MRONJ onset. The progression of MRONJ represents reduced level of bone turnover markers; therefore, some reports state that the CTX value can be used to assess the MRONJ risk or severity [11, 19]. In contrast, several reports have revealed that no biological marker can predict the development and reflect the severity of MRONJ [21]. Marx et al. [11] suggested that the relative risk could be evaluated as follows: CTX values < 100 pg/mL represent high risk, CTX values of 100–150 pg/mL represent moderate risk, and CTX values > 150 pg/mL represent minimal risk. In this study, the average CTX value (219 pg/mL) was higher in the oral BP group than in the IV BP group (134 pg/mL). This suggests that the osteoclast activity and turnover rate of the bone were lower in the IV BP group as compared to that in the oral BP group, indicating that poor prognosis.

ESR is a type of blood test that measures how quickly erythrocytes settle at the bottom of a test tube. Generally, red blood cells settle down relatively slowly. A faster-than-normal rate, and thus elevated ESR, may indicate inflammation in the body. The CRP level rises when there is inflammation throughout the body. It is a group of proteins called acute phase reactants that rise in response to inflammation. Choi et al. [19] reported that the inflammatory markers, ESR and CRP, were significantly higher in MRONJ patients than in the controls and were closely related to MRONJ severity at the time of the diagnosis. MRONJ could cause chronic inflammation; thus, the ESR was elevated in both, the oral and IV administration groups. In the IV group, the CRP level was higher; thus, acute inflammation was more severe than that in the oral BP group.

One of the limitations of this study is the lack of data on other drugs taken with BP, such as corticosteroids, and close examination of the patients’ medical history. Further research is required to determine the difference in the doses for oral and IV BP medication that can cause MRONJ.

In conclusion, IV administration of BP causes more severe inhibition of bone remodeling and could result in a higher degree of inflammation. Therefore, even if the duration of IV medication of BP is shorter than that of oral BP, the extent of lesion could be more extensive. Furthermore, IV BP is administered for MRONJ is associated with poorer prognosis.

## Data Availability

The datasets used and/or analyzed during the current study are available from the corresponding author on reasonable request.

## Abbreviations

BP: Bisphosphonates
IV: Intravenous
MRONJ: Medication-related osteonecrosis of the jaw
ONJ: Osteonecrosis of the jaw
AAOMS: American Association of Oral and Maxillofacial Surgeons
CTX: C-telopeptide of collagen type 1
ESR: Erythrocyte sedimentation rate
CRP: C-reactive protein
